# A cyber-linked undergraduate research experience in computational biomolecular structure prediction and design

**DOI:** 10.1371/journal.pcbi.1005837

**Published:** 2017-12-07

**Authors:** Rebecca F. Alford, Andrew Leaver-Fay, Lynda Gonzales, Erin L. Dolan, Jeffrey J. Gray

**Affiliations:** 1 Department of Chemical and Biomolecular Engineering, Johns Hopkins University, Baltimore, Maryland, United States of America; 2 Department of Biochemistry and Biophysics, University of North Carolina at Chapel Hill, Chapel Hill, North Carolina, United States of America; 3 Texas Institute for Discovery Education in Science, University of Texas, Austin, Texas, United States of America; 4 Department of Biochemistry and Molecular Biology, University of Georgia, Athens, Georgia, United States of America; 5 Institute for NanoBioTechnology, Johns Hopkins University, Baltimore, Maryland, United States of America; Genome Quebec, CANADA

## Abstract

Computational biology is an interdisciplinary field, and many computational biology research projects involve distributed teams of scientists. To accomplish their work, these teams must overcome both disciplinary and geographic barriers. Introducing new training paradigms is one way to facilitate research progress in computational biology. Here, we describe a new undergraduate program in biomolecular structure prediction and design in which students conduct research at labs located at geographically-distributed institutions while remaining connected through an online community. This 10-week summer program begins with one week of training on computational biology methods development, transitions to eight weeks of research, and culminates in one week at the Rosetta annual conference. To date, two cohorts of students have participated, tackling research topics including vaccine design, enzyme design, protein-based materials, glycoprotein modeling, crowd-sourced science, RNA processing, hydrogen bond networks, and amyloid formation. Students in the program report outcomes comparable to students who participate in similar in-person programs. These outcomes include the development of a sense of community and increases in their scientific self-efficacy, scientific identity, and science values, all predictors of continuing in a science research career. Furthermore, the program attracted students from diverse backgrounds, which demonstrates the potential of this approach to broaden the participation of young scientists from backgrounds traditionally underrepresented in computational biology.

## Author summary

Computational biology research is frequently conducted by virtual teams: groups of scientists in different locations that use shared resources and online communication tools to collaborate on a problem. It is imperative that the next generation of computational biologists can easily work in these interdisciplinary, distributed settings. However, most undergraduate research training programs are hosted by a single institution. In this report, we describe a new summer undergraduate research program in which students conduct biomolecular modeling research with the Rosetta software in research groups around the world. The students each conducted their own research project in a university-based group while collaborating with other students and members of the Rosetta Commons at a distance using everyday tools such as Slack, Skype, GitHub, and Google Hangouts. When compared with in-person summer research training programs, students report similar or even improved outcomes, including the development of a sense of community and increases in their scientific self-efficacy, scientific identity, and science values. Furthermore, our program attracts a diverse group of students and thus has the potential to help broaden participation in computational biology.

This is a *PLOS Computational Biology* Education paper.

## Introduction

Computational biology is an interdisciplinary field, and many computational biology research projects are performed by distributed international teams of scientists. In the coming decade, it will be imperative for computational biologists to collaborate within these virtual communities [1,2]. However, few undergraduate programs expose students to a distributed research environment. Introducing new training paradigms is one way to facilitate research progress in computational biology. In this work, we describe the Rosetta Research Experience for Undergraduates (REU), a program in biomolecular structure prediction and design in which students conduct research in a distributed environment. We detail the structure of the program designed to expose students to a virtual community and describe student research experiences from the first two cohorts.

Undergraduate research experiences are important avenues for recruiting and preparing the next generation of scientists [3]. Hands-on lab experiences encourage creativity and expose students to problem-solving frameworks [4]. Students who spend significant time in the lab learn to perform new techniques, collect data, interpret findings, and formulate new research questions [5,6]. Lab experiences can shape students’ perceptions about careers in research [7]. Through undergraduate research experiences, students gain access to professional mentors who provide career support needed to retain a diverse group of students in science and engineering. Undergraduate research can also serve as an introduction to fields such as computational biology, which are not well represented in undergraduate degree programs or courses, especially at institutions that serve large proportions of students from underrepresented backgrounds.

In the United States, REU sites are funded by the US National Science Foundation (NSF) and serve as a major mechanism for involving undergraduates in science research. Most REU sites offer 10-week summer programs designed to engage 8–10 undergraduates in meaningful research [8] and to recruit students, especially those from underrepresented backgrounds, into graduate education and research-related careers [9]. Students participate in hands-on lab or field research experiences, complemented by journal clubs, sessions for writing and presentation peer review, and information sessions about graduate education and research-related career options. In general, REU sites are hosted by a single department, program, center, or institution.

This REU structure is inherently limiting for computational biology because computational biology research is performed by geographically-distributed teams of scientists with varied academic backgrounds ranging from mathematics and computer science to cellular and molecular biology. In addition, scientific projects depend on shared computing resources, data sets, and codebases. To be successful in computational biology, students need to develop interdisciplinary research skills such as the ability to formulate integrative research questions and communicate with researchers in other fields [10]. These distinctions require rethinking how to structure REUs to meet the unique needs and challenges of computational biology.

We created a new REU program within the Rosetta Commons, a group formed to enable close collaboration between 52 (and growing) labs developing the Rosetta software suite for biomolecular structure prediction and design. The Rosetta Commons labs are united by a set of core challenges, including (1) sampling macromolecular conformational space, (2) improving energy functions, (3) utilizing advanced computing resources, (4) improving code organization and algorithm efficiency, and (5) disseminating the tools to academic and industry labs. To tackle these challenges, community developers from a broad range of fields have contributed tens of thousands of revisions to the master version of Rosetta from their development branches. Collaborating scientists have tackled a wide range of science and engineering challenges, from RNA folding [11] to the refinement of structures using NMR data [12] to designed proteins [13,14], interfaces [15–17], protein nanomaterials [18,19], mineral binders [20], and antibodies [21,22]. The public has also engaged in Rosetta-mediated science through the Berkeley Open Infrastructure for Network Computing (BOINC)-distributed computing platform [23] and game-playing applications such as Foldit [24].

The Rosetta collaboration is an appropriate environment for a geographically-distributed computational biology REU for two key reasons. First, the problem-solving approaches are highly interdisciplinary. For instance, X-ray crystallography and NMR were originally developed in physics and chemistry, and sequencing and protein expression originated in biology. Second, labs at different institutions are already connected by online communication tools. In particular, the GitHub code-sharing platform [25], Slack team messaging [26], and an in-house benchmarking server allow developers to work on a common source in their own branch, request code review, tag collaborators, comment on developments, and easily share their work.

In this report, we describe the implementation and evaluation of the Rosetta biomolecular modeling REU, the first REU situated within a globally distributed scientific community. We describe our strategies for recruiting a diverse cohort of students and explain the implementation of the three program phases: (1) one week of intensive, hands-on learning about computational methods development, (2) eight weeks of research at different Rosetta labs, and (3) one week at the Rosetta annual conference. We discuss strategies we used to keep students connected while they conducted their research. We describe early evaluations of the program and student outcomes. Finally, we discuss the program goals as they align with grand challenges in undergraduate science education, and we postulate next developments therein.

## Student recruitment and selection

### Recruiting a diverse cohort of students

A primary goal of the Rosetta REU was to attract and retain underrepresented groups in computational science, chemistry, engineering, and the biosciences. We took a two-pronged approach to recruit a diverse cohort. First, we promoted the program via email to several organizations, including the Society of Women Engineers (SWE), Hispanic Association of Colleges and Universities (HACU), the Society of Hispanic Professional Engineers (SHPE), the National Society of Black Engineers (NSBE), and the American Indian Science and Engineering Society (AISES). We reached out via email to local universities with diverse populations. We also partnered with diversity programs, including Minority Access to Research Careers (MARC) and the Leadership Alliance, by asking them to distribute the program information and recommend potential participants.

Second, we reached out to attendees at two affinity group conferences. For the last three years, we have sent a delegation of two faculty plus 6 to 10 female scientists from multiple Rosetta labs to the Grace Hopper Celebration of Women in Computing. The two faculty led a Student Opportunity Lab round-table to present “Computational Molecular Biophysics: Design Your Future.” In addition, the delegation hosted a booth at the career exposition with demonstrations and information. At this event, we collected over 40 resumes annually and eventually recruited three students through this outreach. We recently replicated this effort with an initiative to minority students by attending the Annual Biomedical Research Conference for Minority Students (ABRCMS). At the conference, we collected between 40 and 60 resumes and followed up with these students, encouraging them to apply for the program via email, eventually enrolling one program participant.

### Application and student selection

The program was open to all undergraduate science, mathematics, and engineering students who had not graduated before the summer session. To apply, students submitted an online application that included a personal statement, summary of research and computing experience, resume, transcript, lab assignment preferences, and contacts for three reference letters. In the personal statement, students were asked to explain why they are interested in the REU program and how the projects fit with their interests and talents. The experience statement required students to summarize their academic achievements, special skills, academic honors, and other creative work.

We sought both computer science majors with no previous biology experience and life science majors with wet lab experience but limited computational background. Previous experience was not required but preferred, to increase the likelihood of student success in the program. The applications were evaluated by a panel of two professors and two graduate students. The criteria for evaluating applications are detailed in S1 File. After selection, we contacted students to confirm their interest, and then we asked the student and the assigned faculty to meet via Skype to discuss project ideas and again confirm their interest in working together.

## Structure of the research experience

### Week 1: Rosetta Boot Camp

To provide students with a foundation in computational methods development, we initiated the program with one week of hands-on practice at Rosetta Boot Camp [27]. Rosetta Boot Camp is an in-person workshop designed to teach software development skills and Rosetta3 library [28] concepts to new graduate students and postdoctoral fellows. We adapted this workshop for undergraduates by emphasizing skills not taught in traditional courses yet necessary to begin research. We also structured the boot camp to achieve a 4:1 student-to-teacher ratio and to promote collaboration between students. A set of detailed learning objectives is listed in S1 File.

**Fig 1:**
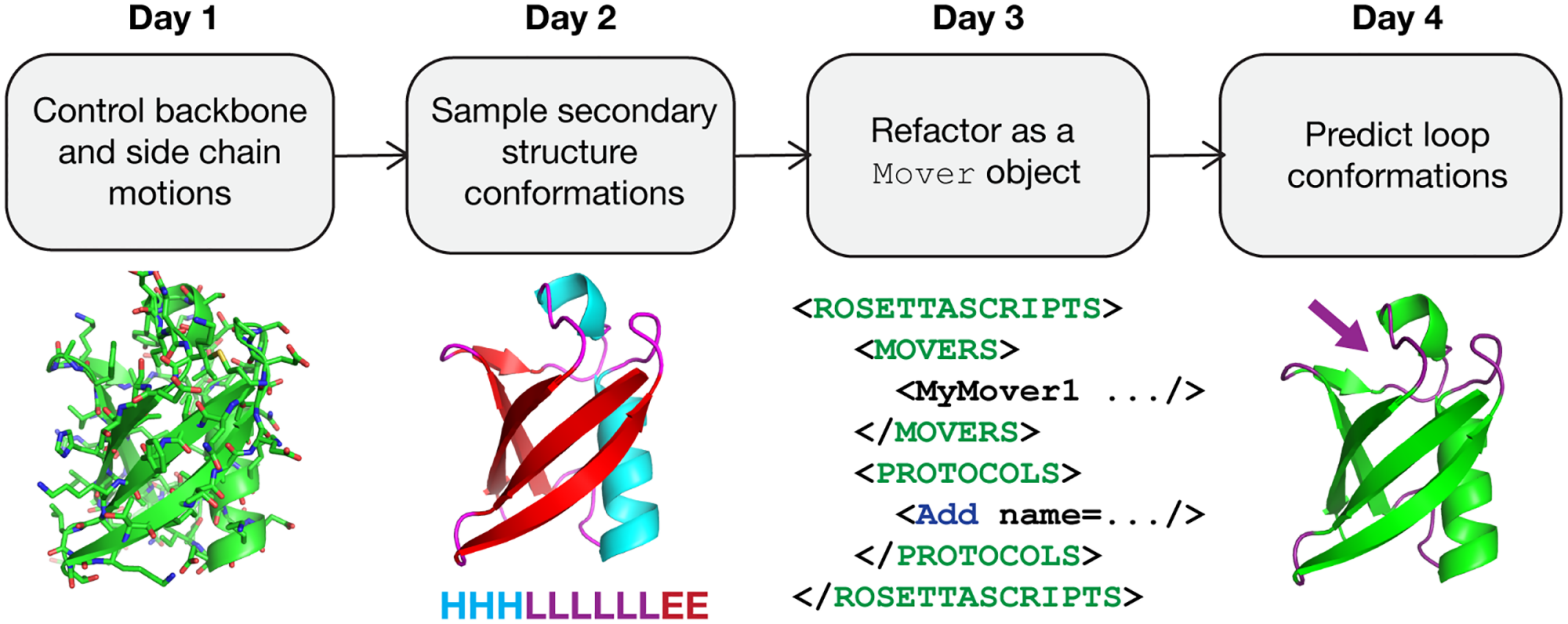
Overview of the “Build your own Rosetta protocol” lab. During the evenings, students worked on a lab activity designed to guide them through the process of writing a Rosetta protocol that takes advantage of different sampling strategies. On Day 1, students outlined a basic Rosetta executable that perturbed structures and then recovered from the perturbation using side-chain packing and whole-structure minimization. On Day 2, students used the FoldTree to restrict the propagation of structural perturbations by partitioning the structure by its secondary structure. On Day 3, students wrapped their protocol in a Mover class that could be hooked into the job distribution system and our XML-based scripting language, RosettaScripts. On Day 4, students applied the CCD method to close loops opened by their perturbations. Day 5 was unstructured time for students to complete their labs. CCD, cyclic-coordinate-descent.

To achieve the learning objectives, students participated in a combination of lecture and lab activities. First, interactive lectures were used to introduce concepts (Table 1). Then, students collaboratively worked on two types of activities (Table 2). The first set focused on skills needed to write, test, debug, and version-control code. The second set (marked by an asterisk in Table 2) walked students through the creation of a complex conformational sampling protocol. In the first lab, they wrote an application to perturb and minimize a structure using core Rosetta modules. In subsequent labs, they refined this protocol to more carefully control how perturbation propagated through the structure, dividing structures by secondary structure elements, and eventually incorporating the cyclic-coordinate-descent (CCD) loop-closure algorithm [29] to improve the likelihood that perturbations would result in low-energy conformations. They connected their protocol to the job-distributor machinery in Rosetta and to RosettaScripts: two parts of Rosetta that many students would work with during their internships (Fig 1).

**Table 1:** Overview of Rosetta Boot Camp lecture topics.

Day	Lecture Topic	Learning Objectives
Monday	Introduction to computational protein structure prediction and design	--
	Introduction to the C++ programming language	1.a.i, 1.a.ii
Tuesday	Utility, numeric, basic, and core Rosetta3 libraries	2.a.i, 2.a.ii, 2.a.iii
	Core Rosetta3 libraries	2.a.i, 2.a.ii, 2.a.iii
Wednesday	Writing protocols in RosettaScripts	2.e.i, 2.e.ii, 2.e.iii. 2.e.iv, 3.e.i, 3.e.ii, 3.e.iii, 3.e.iv, 3.e.v
	Const correctness in C++	2.d.iv, 2.d.v
Thursday	Common Rosetta modeling protocols	2.c.i, 2.c.ii, 2.c.iii, 2.c.iv, 2.c.v, 2.c.vi, 2.c.vii
	Controlling flexibility during modeling	3.f.ii.4
Friday	Adding code to Rosetta	3.f.i, 3.f.ii, 3.f.iii, 3.f.iv, 3.f.v, 3.f.vi, 3.f.vii, 3.f.viii, 2.b.i, 2.b.ii, 2.b.iii

**Table 2:** Overview of Rosetta Boot Camp lab activities.

Day	Lab Activities	Learning Objectives
Monday	Version control and branching with Git	1.c.i, 1.c.ii, 1.c.iii, 1.c.iv, 1.c.v, 1.c.vi, 1.c.vii
	Writing your first Rosetta C++ modeling protocol*	2.d.i, 2.d.ii, 2.d.iii, 2.e, 2.f.i, 2.f.ii.1, 2.f.ii.2, 3.c, 3.a.i, 3.a.iii, 3.a.v
Tuesday	Writing unit tests for C++ classes	3.a.ii, 3.a.iv, 3.b.i, 3.b.ii. 3.b.iii, 3.b.iv, 3.b.v, 3.b.vi
	Kinematic control with the FoldTree*	2.f.ii.3, 3.d
Wednesday	Writing a protocol in RosettaScripts	3.e.i, 3.e.ii, 3.e.iii, 3.e.iv, 3.e.v
	Packaging protocols in a Mover subclass*	1.d.i, 1.d.ii, 1.d.iii, 1.d.iv
Thursday	Unix primer and scripting with bash, sed, and awk	1.a.iii, 3.d
	Loop modeling with CCD*	2.f.ii.4
Friday	Extra time to complete remaining labs	--

*Each lab builds on the previous lab marked with an asterisk toward development of a complex modeling protocol.

The workshop was led by a primary instructor and two student teaching assistants, including alumni of the program and a student volunteer from the Rosetta community. Students prepared by completing readings and short C++ homework assignments. During the week, students worked in groups on the lab activities to encourage sharing of complementary knowledge. This was crucial because both cohorts comprised students with diverse academic backgrounds. Finally, we assessed the students’ progress through code review, short-answer concept tests, and assignment completion.

### Weeks 2–9: Research in labs

Over the next eight weeks, each student conducted a research project in one of the 52 Rosetta Commons labs, typically under the supervision of a senior graduate or postdoctoral researcher in the lab. The students remained connected with each other and other participating research groups through several channels discussed below.

#### Main Rosetta developer channels

The students joined several platforms typically used for collaboration within the Rosetta Commons. First, students joined the Rosetta Slack team to directly ask developers about code design, debugging strategies, and scientific approaches in real time. In addition, students joined the Rosetta GitHub team to participate in online code reviews and track contributions to the codebase. Finally, students were given access to our custom benchmark server, which enables us to test code changes.

#### Virtual journal clubs

To connect the cohort scientifically, we held a virtual journal club each week. The meeting occurred via Zoom video conference so that all participating students and two faculty members were connected. Two students presented each week, such that each student presented twice during the summer. For the first presentation, students were asked to explain a paper published by their host lab. The assignment provided students with the opportunity to learn the science of their host lab in detail and share it with their program peers. For the second presentation, students chose a paper from the wider literature. Each faculty member cohosted one or two of the journal clubs during the summer (typically not the same week their mentee presented). The faculty members facilitated the discussion, ensuring that each student participated, encouraging in-depth understanding, ensuring that questions were answered, and facilitating broader brainstorming about the potential impacts and future directions of the work.

#### Writing and presentation skill development

Written and oral communication skills are critical for science and engineering research. To maximize scientific exchange in the cohort, we held peer critiques of writing during the summer. During week five, students wrote a two-page proposal describing their summer research following the format of the NSF Graduate Research Fellowship application [30]. In addition, students drafted scientific posters for the Rosetta conference in week nine. For both activities, students were paired up across different labs to exchange proposals for critiquing, and they also received feedback from their host lab mentors.

#### On-site partnerships with local REU cohorts

To enable students to build a local network of peers and more experienced scientists, we formed partnerships with summer programs at all participating institutions. Many of these programs included social activities (e.g., brown bag lunches, picnics, outings to museums), professional development (e.g., networking sessions, discussions on relevant topics such as graduate education, work–life balance, career options), mock interviews with PhD admissions directors, and lunch seminars with visitors from academia and industry.

### Week 10: The annual Rosetta conference (“RosettaCon”)

Each summer, the Rosetta Commons members convene to discuss the newest science to emerge from the collaboration. This meeting is held in Washington state and involves about 250 people from the 52 Rosetta labs, plus invited speakers. The first two days are held on the University of Washington campus and are meant to facilitate discussion on software and ongoing technical challenges. The following three days occur at the Sleeping Lady Conference Center in Leavenworth, Washington, and consist of scientific presentations, small group discussion, posters, and leadership and team meetings.

Students attended the full conference, which allowed them to reconnect with one another in person, network with other researchers at the conference, and learn about the wider field of computational biology. Each student presented a poster of their research accomplishments and received feedback on their work. Finally, we held a debriefing session for the cohort in which we solicited feedback about the program.

## Results

### Description of the first two cohorts

We hosted eight interns during the summer of 2015 and eight interns during the summer of 2016 in 14 different Rosetta Commons labs. We also educated a diverse cohort of students: across both cohorts, 63% of students were female, 13% were African American, and 13% were Hispanic. The students conducted a diverse set of scientific projects described in Table 3.

**Table 3:** Intern projects from the summer 2015 and summer 2016 cohorts.

Cohort	Project	Principal Investigator	Institution	Location
2015	Redesigning HIV broadly neutralizing antibody PGT 121 to maintain stability and increase binding potency	Bill Schief	Scripps Research Institute	La Jolla, CA
2015	Encoding covariation into re-design of PDZ domains: Is sequence tolerance context-independent?	Tanja Kortemme	University of California at San Francisco	San Francisco, CA
2015	Quantification of local contact densities at protein-small molecule and protein-protein interfaces	Justin Siegel	University of California at Davis	Davis, CA
2015	Stepwise redesign: Application for designing atomic resolution RNA	Rhiju Das	Stanford University	Stanford, CA
2015	Marburg virus antibody modeling using comparative modeling	Jens Meiler	Vanderbilt University	Nashville, TN
2015	Carbohydrate and protein effects on antibody-receptor binding	Jeffrey Gray	Johns Hopkins University	Baltimore, MD
2015	Scoring sequence for modeled folding conformation in InteractiveROSETTA using a Hidden Markov Model based on sequence-structure motifs	Chris Bystroff	Rensaleer Polytechnic Institute	Troy, NY
2015	Analyzing the molecular interactions of the α-GID/α4β2 receptor complex: An evaluation for drug design	Richard Bonneau	New York University	New York, NY
2016	Iteratively building hydrogen bond networks at protein-protein interfaces	Brian Kuhlman	University of North Carolina at Chapel Hill	Chapel Hill, NC
2016	Ligand Holes: Screening for better fitting ligands	John Karanicolas	University of Kansas	Lawrence, KS
2016	Improving player onboarding in citizen science games with three-star systems	Seth Cooper	Northeastern University	Boston, MA
2016	Computational design of auto-inhibited chemotherapeutic enzyme using Rosetta	Sagar Khare	Rutgers University	New Brunswick, NJ
2016	Structure-based prediction of non-histone human deacetylase (HDAC) 2 substrates	Ora Schueler-Furman	Hebrew University	Jerusalem, Israel
2016	Modeling cancerous mutations in CCCTC binding factor “Core”	Richard Bonneau	New York University	New York, NY
2016	Predicting glycoforms of Mucin 1 in cancer cells and identifying their binding forms	Jeffrey Gray	Johns Hopkins University	Baltimore, MD
2016	Computational design of co-assembling multi-component protein crystals in the F222 space group	David Baker	University of Washington	Seattle, WA

### Student research achievements

Rosetta REU students have already shared their work with the scientific community in the format of formal presentations and publications. All students shared the outcomes of their scientific projects at the Rosetta conference. Two students have presented their work at other scientific meetings, and one student is an author on a conference paper [31,32]. In addition, two students contributed code to the main Rosetta repository; their contributions are already being distributed to end users. These scientific deliverables demonstrate that students can conduct high-level research projects in the eight-week time span.

Informally, we observed that the interns helped to advance the research of the host lab. For example, one intern used a newly developed framework for modeling protein glycosylation [33] to create models of antibody constant regions with different mutations and glycosylations that affect binding to antibody receptors and immune stimulation [31]; this work continues in the host lab and has enabled new collaborations with experimental labs. Another intern examined the computer–human interface for the protein-folding game FoldIt [24] to measure how three-star rating systems affect game player persistence [32]. One student designed co-assembling multicomponent protein crystals, and the host lab invited him back for a second summer to continue the research.

### Student career progress

Most of the students who participated in the REU program are now pursuing careers in science. Of the twelve alumni who have completed their BS degree, six students are now PhD candidates in fields ranging from chemical engineering to computer science and molecular biology. Two are working in the pharmaceutical industry, one is working in an academic research lab, and one is working as a high school mathematics teacher. One is currently applying to medical school, and three from the 2016 cohort are currently applying to graduate school (as of fall 2017).

### Evaluation of virtual cohort structure

To evaluate our virtual REU model, we surveyed both cohorts of students at the end of each summer about their sense of community, scientific self-efficacy, scientific identity, and the extent to which their personal values aligned with scientific values [34–36]. These outcomes are indicators of the students’ integration into their scientific community and predictors of their likelihood to continue in science research–related career paths, especially for students from backgrounds traditionally underrepresented in the sciences [35]. We compared the responses of our students with responses from students in two in-person, computational life science REU programs.

**Fig 2:**
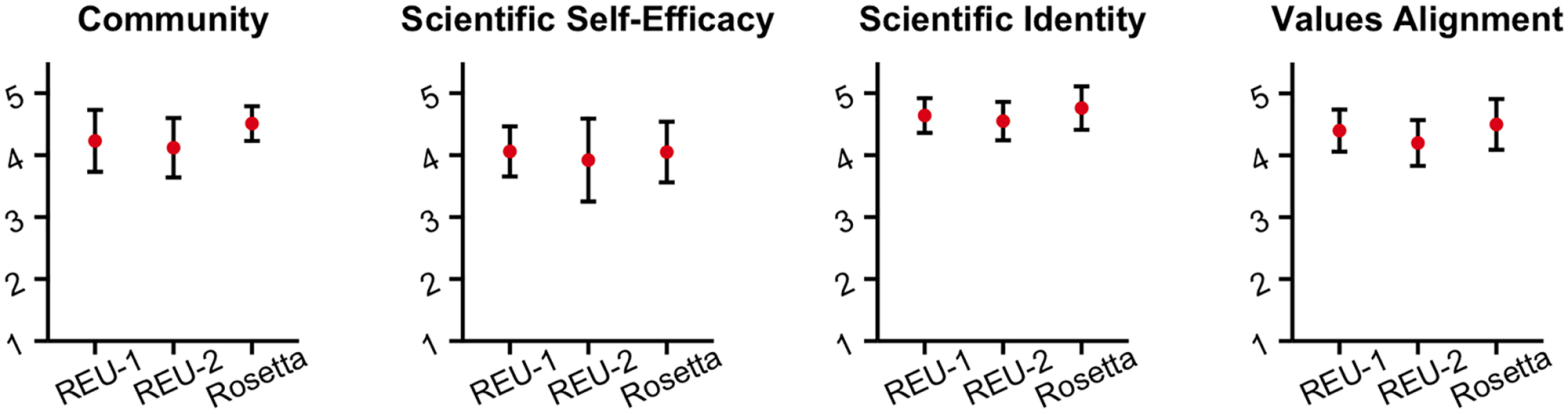
Comparison between the Rosetta REU and two other life science REU programs. We surveyed students at the completion of the program on four outcomes: sense of community, scientific self-efficacy, scientific identity, and values alignment. Here, these data are compared to the survey results of two other life sciences REU programs. REU, Research Experience for Undergraduates.

Post-program survey data (Fig 2) show that both cohorts matched the “sense of community” of other programs. Interview comments reinforce the strong community even across distributed virtually-linked labs (see S1 File). Similarly, the data revealed that our program matched outcomes for scientific self-efficacy, scientific identity, scientific values alignment, and their intentions to pursue a science research–related career.

## Discussion

In this report, we presented a summer research experience that involves undergraduates in distributed computational biology research. We also attracted a diverse cohort, demonstrating the potential of this approach to broaden participation by students from traditionally underrepresented backgrounds. After the first two cohorts, we pooled our experiences to identify strengths and weaknesses in the program. Here, we elaborate on these takeaways and recommend directions for improvement.

### Introducing students to an interdisciplinary field at boot camp

A primary challenge of our program was teaching students with varied academic backgrounds. Most undergraduate science programs do not include quantitative courses beyond prerequisite calculus [37]. Furthermore, computational biology degree programs are still new [38] and seldom available at institutions that primarily serve students from underrepresented backgrounds. Therefore, we anticipated that students would vary in their preparation to do computational work.

At boot camp, we prepared to support students with a high instructor-to-student ratio (1:4). We also arranged the students around a conference table intended to facilitate collaboration while working on lab activities. One hurdle was teaching the Unix command line because half of the students had no prior experience. This knowledge is critical because most molecular modeling programs are controlled from the command line. Initially, we tried to pair students with and without experience. However, we found that the more experienced student felt held back. In the future, we plan to include more Unix preparation in the homework preceding boot camp. We also hope to integrate strategies that encourage patience when working in teams with mixed backgrounds.

For future work, we also plan to further develop the boot camp learning objectives (see S1 File). Undergraduate boot camp was derived from a workshop intended for new graduate students and postdoctoral fellows. Thus, the week is packed with technical details about C++ language features and the mathematics underlying Rosetta algorithms. However, we postulate that skills required for an eight-week internship may differ. For instance, students are more likely to apply the tools and analyze results rather than develop new protocols from scratch. Furthermore, undergraduates may benefit from developing more transferable skills. In the future, we plan to revisit the objectives and potentially rebalance toward more general computational biology skills rather than those specific to Rosetta.

### Encouraging students to leverage collaboration tools

The Rosetta REU program is a “proof of principle” example that undergraduates can perform research in a distributed setting. We found that students made strong connections within the cohort that matured into an internal collaboration network during the eight-week research period. A few students even contributed code and commented on ongoing projects via the GitHub [25] code-sharing platform. All these findings are reinforced by survey reports that students experienced a strong sense of community.

Forming strong bonds between students is a top priority of the program. As the program continues, we are aiming to help mentors better guide and connect with their students during the eight-week research period by drawing more from evidence-based mentoring practices [39–41], and we want students to leverage weak ties [42] in the Rosetta community. Students were given access to several collaboration tools, including the Slack [26] channel and developer mailing lists. However, we observed that the students used these tools sparingly. In scientific communities, weak ties are critical because reaching out of one’s inner network increases the probability that knowledge transfers are more novel. One possibility of encouraging students would be to scaffold using community resources during boot camp rather than introducing them at the end. This way, students can begin using the tools under instructor guidance, gain confidence, and then apply them.

### Attract and retain underrepresented groups in computational sciences

Another goal of the Rosetta REU program was to foster an inclusive culture. Diversity is critical to the creativity and productivity of teams [43]; however, recruiting a diverse cohort remains a challenge, especially in computer science and mathematics [44]. To address this goal, we attended affinity conferences and reached out to affinity groups, thereby adding more applications to our pool. Sending student and faculty representatives to these conferences also allowed our students and faculty to learn strategies to confront the confidence gap [45] and unconscious bias [46]. Overall, this also increases awareness of these issues not only within our small group but also amongst the larger Rosetta community.

We postulate that the diversity of the REU cohort also contributed to the strong sense of community. In addition, our recruiting efforts at Grace Hopper and ABRCMS strengthened our community of women in the Rosetta Commons, and by rotating the attending faculty, more received education and awareness of gender issues in the field. Upon returning to the labs, these conference delegates have sparked other diversity efforts, including broader conference activities, Lean In Circles [47], and monitoring of conference speaker diversity. In the future, we will continue to engage in affinity conferences and take home new practices for fostering and encouraging diversity and inclusiveness in virtual cohorts and the Rosetta community overall.

## Supporting information

S1 File Includes student selection criteria, Rosetta Boot Camp learning objectives, selected responses from student surveys, and contact information for the program director and coordinator. (PDF)
