# Supplementary material for: A cyber-linked undergraduate research experience in computational biomolecular structure prediction and design
Source: PLoS Comput Biol. 2017 Dec 7;13(12):e1005837. doi: 10.1371/journal.pcbi.1005837 (PMC5720517; doi:10.1371/journal.pcbi.1005837)
Supplement: S1 File — (PDF) [file pcbi.1005837.s001.pdf]

Supporting Information

"A cyber-linked undergraduate research experience in biomolecular structure prediction and design"

Rebecca F. Alford, Andrew Leaver-Fay, Lynda Gonzales, Erin L. Dolan, Jeffrey J. Gray

Student selection criteria **2**

Rosetta Boot Camp Learning objectives **3**

Selected responses from student survey **6**
Comments about the research community *6*
Regarding program outcomes *6*

Contact information for program director **7**

Student selection criteria

Student applications were evaluated using the following criteria. Specifically, we ranked each application on a scale of one to five, with:

- 1 = Stellar, must-have student
- 2 = Super, student would be good for the program
- 3 = Acceptable for the program
- 4 = Likely not a good match
- 5 = Not a good match

Table S1. Student selection criteria

Criteria	Description
Potential for graduate school	The student has demonstrated an interest in graduate school in their essay
Potential for scientific research	The student has previous experience working on a scientific research project in an academic or industry lab
Potential in computer programming	The student has previous experience or has taken one or more courses in computer programming
Potential in biophysics and biochemistry	The student has previous experience or has taken one or more courses in biophysics and/or biochemistry
Motivation	In the essay, the student has demonstrated interest and enthusiasm for the field of computational biology and biophysics
Strength of recommendations	Letters of recommendation indicate the student is motivated to learn and has the potential to succeed in a research lab
GPA and transcript	Transcript demonstrates strong academic performance in relevant courses
Match quality to an open project	The students' prior experience and skills are well matched to an available project. The student also indicated interest in that project.
Contribution to diversifying our community	The student is part of a group traditionally underrepresented in science and engineering.

Rosetta Boot Camp Learning objectives

Here we list learning objectives for Rosetta Boot Camp: a one-week workshop designed to orient students to software engineering and biomolecular modeling in Rosetta. The objectives are organized into three categories: (1) development of translational software engineering skills, (2) navigation of the Rosetta 3 software suite and (3) creation of new methods using the Rosetta 3 libraries.

1. Develop translational software engineering skills
    a. Interact with a computer using the Linux/Unix command line
        i. Open files from the command line
        ii. Navigate directory hierarchies with `cd`
        iii. Search for code using `ack` (`ack-grep`)
    b. Write unit tests using the CxxTest suite
        i. Describe the purpose of a unit test
        ii. Define code correctness
        iii. Develop code that is 100% testable (a "test first" strategy)
        iv. Add, compile, and run a new unit test suite for new code
        v. Add a new unit test to an existing suite
        vi. Write comprehensive tests for a small class
    c. Manage software development using Git and GitHub for version control
        i. Summarize the benefits of version control
        ii. Create and manage branches
        iii. Stage files for commits
        iv. Commit changes to a repository
        v. Merge and update branches
        vi. Resolve conflicts that arise upon merging branches
        vii. Identify a reference for learning advanced Git commands as needed
    d. Write object-oriented C++ programs
        i. Write a method in C++
        ii. Write an overloaded method in C++
        iii. Write a class in C++
        iv. Apply C++ specific keywords: `const`, `this`, `static`
        v. Explain the benefits and limitations of smart pointers
    e. Gain familiarity with object-oriented programming
        i. Define and summarize the benefits of object-oriented programming
        ii. Recognize frequently used "design patterns" employed in Rosetta: Factory method, Abstract factory, Singleton, Command, Curiously recurring template pattern
        iii. Recognize when encapsulation (private data members) is necessary and why
        iv. Recognize the value provided by virtual functions
2. Navigate the Rosetta 3 object-oriented software suite
    a. Find and interpret Rosetta C++ code
        i. Recognize the location of code given the namespace/directory convention
        ii. Identify documentation for classes and methods from header files
        iii. Identify method details from source files

b. Use key resources for collaborating on Rosetta development
    i. Access and ask questions on the Slack channel
    ii. Access and ask questions on the Rosetta developer email list
    iii. Access and ask questions on the Rosetta forums
c. Know the purpose of frequently used Rosetta protocols
    i. *ab initio* protein structure prediction via fragment insertion
    ii. Protein design via the pack_rotamers module
    iii. High-resolution structure refinement with the relax protocol
    iv. Docking as a combination low-res / high-res protocol
    v. Active-site grafting with 6D hashing in the Matcher
    vi. Loop-grafting with 6D hashing in LoopHash
    vii. Coarse backbone "designability" score using 6D hashing in MotifHash
d. Know the data and methods associated with frequently used classes
    i. Describe key methods in a `Mover` class
    ii. Describe key methods in a `Filter` class
    iii. Describe key methods in a `ScoreFunction` class
e. Watch conformational sampling in real time using `PyMOL` and the `PyMOLObserver`
f. Describe conformational sampling strategies using internal geometry
    i. Define downstream coordinate change propagation. List benefits and drawbacks.
    ii. Describe common sampling strategies used by Rosetta:
        1. Control side chain conformations with the packer
        2. Control backbone conformations with `set_phi` and `set_psi`
        3. Control backbone conformations with the `FoldTree`
        4. Close loops using the CCD loop closure algorithm

3. Create new methods in the Rosetta 3 libraries
    a. Use scons to compile existing and new files
        i. Add new files for compilation by editing a src/*.src.settings file
        ii. Add new unit tests for compilation by editing a test/*.src.settings file
        iii. Compile libraries and applications in debug and release mode
        iv. Compile the unit test suites
        v. Create fast recompilation scripts by copying output from scons
    b. Automate structure-processing pipelines using bash, awk, and sed
    c. Write a basic Rosetta protocol in C++
    d. Write an application that minimizes downstream coordinate propagation using a custom-built FoldTree
    e. Write a Rosetta Script to combine existing modules into a new protocol
        i. List benefits of Rosetta's XML interface
        ii. Edit XML files into a Rosetta-scripts-valid format
        iii. Interpret and address schema-validation failures
        iv. Use the schema to list parameters for Movers/Filters/etc
        v. Iteratively improve a Rosetta Script to expand its functionality
    f. Know the steps for contributing code to the master version of Rosetta
        i. Describe the importance of coding conventions
        ii. Write code that adheres to community coding conventions
        iii. Write and add an integration test for a new application
        iv. Create a pull request using the GitHub interface
        v. Merge a pull request using the GitHub interface

vi. Write user-facing documentation on the Rosetta Gollum wiki
vii. Write in-code documentation using Doxygen tags
viii. Use the benchmark server to run integration and unit tests on a development branch

Selected responses from student survey

Comments about the research community

- “When the interns joined up again at the conference it was like no time had passed”
- “The networking opportunity I had was invaluable. I know contacts all across the country I can go to for advice, and even a letter of recommendation. The fact that I was treated like a graduate student and given essentially free-reign on the project vastly improved my confidence in conducting scientific research”
- “One of the highlights of my experience was how close-knit I became with the other graduate students in the lab”
- “Science I’m the first in my family to go to graduate school, it was really helpful to get to know the faculty mentor and graduate student assistant.”
- “Rosetta was developed by a lot of different labs and there are constantly new things being added, so there has to be some way of managing that process. I learned how a large team can contribute and all maintain the same code base”

Regarding program outcomes

- “I feel more confident in my ability to be more independent in my projects”
- “The research experience helped me learn about my own research and work style, and the improvements I need to make to my work ethic. It really clarified for me what a graduate school experience would look like, and showed me that I really would enjoy doing that. Because of this program I have a renewed motivation to participate in graduate school”
- “I learned so much about graduate school and the process of research from all members of the lab, not just my mentor”
- “It has taught me about the process of applying to graduate school and has really enforced my goals to pursue a PhD”
- I was introduced to a new side of computational biology/bioinformatics, which I am interested in pursuing further as a possible career field”
- “This summer really solidified my resolve to become a scientist”
- “Before the experience I wasn’t confident about graduate school because I didn’t know what kind of degree I wanted. Now, I’m applying for graduate programs related to computational biology or biomedical informatics which I really enjoyed learning about over the summer”

Contact information for program director

Below is the contact information for the program director and coordinator:

Jeffrey J Gray
Program Director
Professor, Chemical & Biomolecular Engineering
Johns Hopkins University
Phone: 1-410-516-3313
Email: jgray@jhu.edu

Camille Mathis
Program Coordinator
Director of Academic Programs, Institute for Nano Biotechnology
Johns Hopkins University
Phone: 1-41-0516-6572
Email: cmathis@jhu.edu
